# High periprosthetic bone mineral density measured in immediate postoperative period may not guarantee less periprosthetic bone loss in the proximal femur after cementless total hip arthroplasty – A retrospective study

**DOI:** 10.1186/s42836-020-0023-3

**Published:** 2020-01-23

**Authors:** Guangtao Fu, Yuanchen Ma, Junxing Liao, Yunlian Xue, Mengyuan Li, Qingtian Li, Zhantao Deng, Qiujian Zheng

**Affiliations:** 1 Division of Orthopedics, Guangdong Provincial People’s Hospital, Guangdong Academy of Medical Sciences, 106, Zhongshaner Road, Yuexiu District, Guangzhou, Guangdong Province People’s Republic of China; 2 Division of Statistics, Guangdong Provincial People’s Hospital, Guangdong Academy of Medical Sciences, Guangzhou, Guangdong Province People’s Republic of China

**Keywords:** Periprosthetic BMD, Total hip arthroplasty, Bone loss, Retrospective study

## Abstract

**Background:**

Total hip arthroplasty is the most common orthopaedic procedure for the end-stage hip diseases. Periprosthetic bone loss is closely related to the increased risk of implant loosening and periprosthetic fractures, but the predictive value of periprosthetic bone mineral density (BMD) measured immediately after surgery has not yet been investigated.

**Methods:**

From April 2015 to October 2017, 64 patients with femoral neck fracture, hip osteoarthritis, femoral head necrosis, or developmental dysplasia of the hip underwent unilateral total hip arthroplasty. Demographic data, bone mineral density of the hip and spine, periprosthetic BMD of 7 Gruen zones, and radiographic parameters measured preoperatively, 1 week, 3 months, and 12 months after surgery were collected. A *p* value < 0.05 was considered to be statistically significant.

**Results:**

Significant decreases of the periprosthetic BMD were found in Gruen zone 1 (− 8.0%; *p* < 0.05), Gruen zone 2 (− 6.3%; *p* < 0.05), Gruen zone 7 (− 8.6%; *p* < 0.05), and total Gruen zone (− 4.7%; *p* < 0.05) in the first postoperative year, compared with the values measured 1 week after surgery. The relationship between the preoperative BMD of the hip/spine and the BMD of Gruen zone 1 and Gruen zone 7 measured 1 week after surgery did not reach statistical significance. The multiple linear regression analysis illustrated that the bone loss in Gruen zone 7 at the end of the follow-up period was negatively affected (β = − 0.703) by the BMD of Gruen zone 7 measured 1 week after surgery, with a R^2^ of 0.486 (*p* < 0.05). Similar results were also found in Gruen zone 1 (β = − 0.448, R^2^ = 0.186; *p* < 0.05).

**Conclusion:**

There were marked decreases in periprosthetic BMD of the proximal femur in the first postoperative year. The predictive values of preoperative BMD of hip and spine on periprosthetic bone loss after THA were limited. Higher periprosthetic BMD measured in immediate postoperative period may not guarantee less periprosthetic bone loss in the proximal femur after cementless THA.

## Introduction

Total hip arthroplasty (THA) is the most common orthopaedic procedure for the end-stage hip diseases. It could effectively restore hip function and improve patients’ quality of life [1]. Over 310,000 THAs were performed among patients aged 45 and over in the USA annually [2]. As one of the major concerns after THA, periprosthetic bone loss is closely related to the increased risk of implant loosening and periprosthetic fractures, as well as being the major risk factor of implant failure and total hip revision [3]. Some statistical studies showed that the decrease in periprosthetic bone mineral density (BMD) was up to 11% 5 years after THA and up to 21.9% 10 years after THA [4, 5]. Friedl et al. [6] found that early medical intervention could significantly decrease the risk of implant failure after cementless THA. Therefore, early identification of such high-risk patients is crucial to decrease periprosthetic bone loss and the related complications.

Although the exact mechanism is not entirely clear, it was proposed that different pathways lead to periprosthetic bone loss [7]. In addition to the well-known particle disease and stress shielding theory, the patient-related factors, such as obesity, specific genetic background, and systematic BMD in the perioperative period, were the risk factors of periprosthetic bone loss [8, 9]. Dual-energy X-ray absorptiometry (DEXA) is a technique used for measuring BMD [10]. Patients with lower preoperative BMD of the hip, spine, and radius showed more accelerated postoperative periprosthetic bone loss than those patients with normal BMD [11]. However, the predictive value of periprosthetic BMD measured immediately after surgery, which shows periprosthetic bone loss immediately after surgery, has not yet been well investigated.

The purpose of this retrospective study was to test early BMD after THA, by which to prevent periprosthetic bone loss. Our hypothesis was that the lower immediate postoperative periprosthetic BMD of the proximal femur was associated with higher periprosthetic bone loss in the first postoperative year.

## Materials and methods

The study was approved by the Institutional Review Board of the Guangdong Provincial People’s Hospital. Informed consents were obtained from all the participants.

From April 2015 to October 2017, 64 patients with femoral neck fracture, hip osteoarthritis, femoral head necrosis, or developmental dysplasia of the hip were admitted into the Guangdong Provincial People’s Hospital, Guangdong, China. The mean age of the patients was 63 years (range: 40 to 75 years). All patients underwent unilateral primary cementless THA. The inclusion criteria were (1) patients who had failed conservative or previous surgical treatment options for a deteriorated hip; (2) patients who continued to have persistent, debilitating hip pain and a significant decrease in the activities of daily living; (3) moderate to severe arthritis; and (4) primary arthroplasty. The exclusion criteria were (1) patients with secondary osteoporosis; (2) a previous surgical history of the operated femur; (3) a previous administration of anti-osteoporosis agents; (4) inflammatory arthritis; (5) diagnosis with periprosthetic fractures or periprosthetic infection during the follow-up period; and (6) absence of *intact* data of demography, BMD measurement, or radiographic parameters in the first postoperative year.

### Surgical procedures

**Fig. 1:**
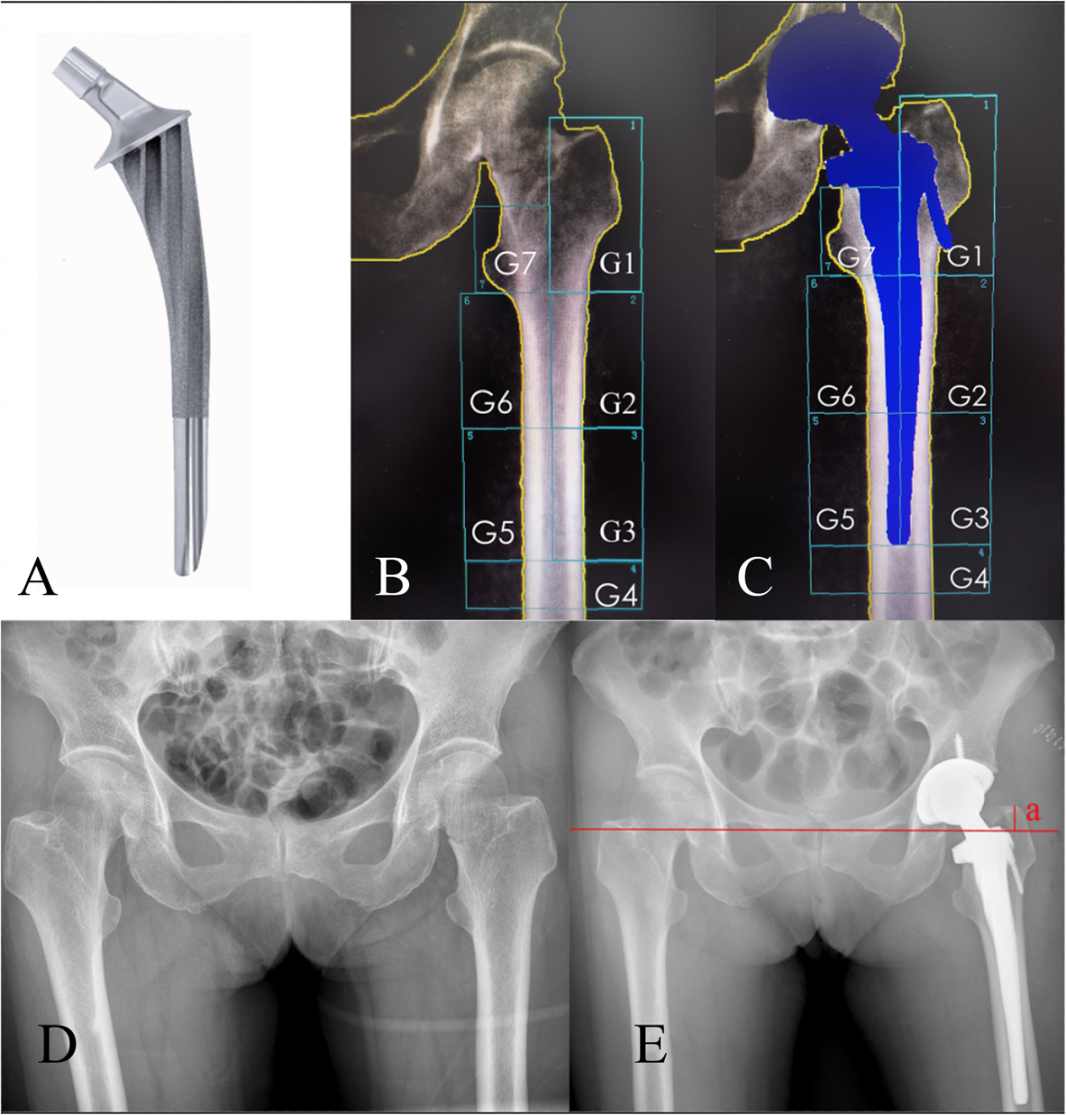
Seven Gruen zones used in Dual-energy X-ray absorptiometrical analysis of preoperative (**a**) and postoperative (**b**) periprosthetic BMD. Preoperative and postoperative anteroposterior X-rays of the pelvic were shown in (**c** and **d**). On anteroposterior X-ray of the pelvis, the inferior displacement of the femoral stem was evaluated by measuring the changes of the distance between the tip of greater trochanter and interteardrop line (Line a in **d**) during the follow-up period (**a**) showsed the appearance of the Ribbed anatomic stem (Waldemar Link GmbH & Co). Seven Gruen zones were used in Dual-energy X-ray absorptiometry analysis of preoperative (**b**) and postoperative (**c**) periprosthetic BMD. Preoperative and postoperative anteroposterior X-rays of the pelvic were shown in (**d** and **e**). On anteroposterior X-ray of the pelvis, the inferior displacement of the femoral stem was evaluated by measuring the changes of the distance between the tip of greater trochanter and interteardrop line (Line a in **e**) during the follow-up period

All operations were performed by one of the three experienced orthopaedic surgeons (QZ, YM, and JL). Operation was performed through the standard posterolateral approach. The femoral prosthesis used in the present study was Ribbed anatomic stem (Waldemar Link GmbH & Co, Germany) (Fig. 1a). The acetabular and femoral components were inserted using the press-fit technique. One week after THA, partial weight bearing was started. Full weight bearing was allowed 2 weeks after THA.

### Data collection

Data of all the participants were retrospectively retrieved from the database of Guangdong Provincial People’s Hospital. Patient demographics (sex, age, height, weight, BMI, primary diagnosis, smoking history, alcohol consumption, type of prosthesis, surgical approach, surgeons who performed the operations, and rehabilitation status) were collected. The BMD of hip and spine, periprosthetic BMD in 7 Gruen zones, and radiographic parameters were also collected preoperatively, 1 week, 3 months, and 12 months after THA.

### Periprosthetic BMD

The BMD of proximal femur and lumbar spine (from L1 to L4) was measured using DEXA (LUNAR DPXMD#5966, Madison, WI, USA). Using DEXA and the associated software, the periprosthetic BMD of the femoral component was analyzed based on the BMD of the seven regions of interest (ROIs), against the protocol proposed by Gruen et al. [12]. Briefly, the proximolateral region was defined as Gruen zone 1, the lateromedial region was defined as Gruen zone 2, and the distolateral region was defined as Gruen zone 3. Correspondingly, the medial periprosthetic region was divided into Gruen zone 5, 6, and 7 from the proximal to distal femur. Gruen zone 4 was located at least 1 cm distal to the tip of the stem (Fig. 1c). The mean total periprosthetic BMD was calculated based on the BMD data of zones 1 to 7 [5]. Preoperatively, the 7 Gruen zones were built with the software, which were comparable to the postoperative measurements of periprosthetic BMD (Fig. 1b). In the present study, the mean least significant changes of the hip, spine, and periprosthetic Gruen zones were 0.017 ± 0.013 g/cm^2^, 0.007 ± 0.005 g/cm^2^, and 0.012 ± 0.015 g/cm^2^, respectively. At the follow-up visit, the inferior displacement of the femoral stem was evaluated by measuring the change of the distance between the tip of the greater trochanter and the interteardrop line on anteroposterior pelvic X-ray (Fig. 1d–e) [13].

### Statistical analysis

All quantitative data were presented as mean ± standard deviation. All values were first assessed using the Kolmogorov-Smirnov test to ensure the normality and Gaussian distribution. Multiple comparisons of changes in periprosthetic BMD between the different time points were analyzed using the analysis of variance for repeated measurements with Bonferroni’s correction. The differences between preoperative and postoperative periprosthetic BMD of 7 Gruen zones were analyzed with Two-sided Student’s *t*-test. The correlations between the preoperative BMD of the hip/spine and the BMD of Gruen zone 1/7 measured 1 week after THA were evaluated with the calculation of the Pearson correlation coefficient. The BMD data of Gruen zone 1 and 7 measured 1 week after THA were also analyzed, resulting in the predictor for the BMD changes of Gruen zone 1 and 7 in the first postoperative year by using a multiple linear regression model with the calculation of the coefficients of determination (R^2^). Other variables enrolled in the multiple linear regression analysis included sex, age, BMI, primary diagnosis, and preoperative BMD of hip/spine. A *p* value < 0.05 was considered to be significant. The SPSS 20.0 (SPSS Headquarters, Chicago, IL, USA) and SAS 9.4 (SAS Institute Inc., Cary, NC, USA) were used to carry out the statistical analysis.

## Results

A total of 64 patients (32 male and 32 female) were enrolled, and the mean follow-up time was 16 months (range from 13 to 22 months). According to the preoperative BMD of hip and spine, 21 patients had T scores above − 1, and the remaining 43 patients had T scores between − 1 and − 2.5 (Table 1).

**Table 1:** Demographic data (mean ± SD)

Patient characteristics	*N* = 64
Sex	
Male	32
Female	32
Age (year)	57.8 ± 12.0
Weight (kg)	64.5 ± 14.0
Height (cm)	163.4 ± 9.9
BMI (kg/m^2^)	24.2 ± 5.3
Smoking history	21
Alcohol consumption	36
Diagnosis	
Femoral head necrosis	48
Femoral neck fracture	8
Hip joint osteoarthritis	5
DDH	3
T score of hip and spine	
T > −1	21
-1 ≥ T ≥ − 2.5	43
-2.5 > T	0

### Periprosthetic BMD

**Fig. 2:**
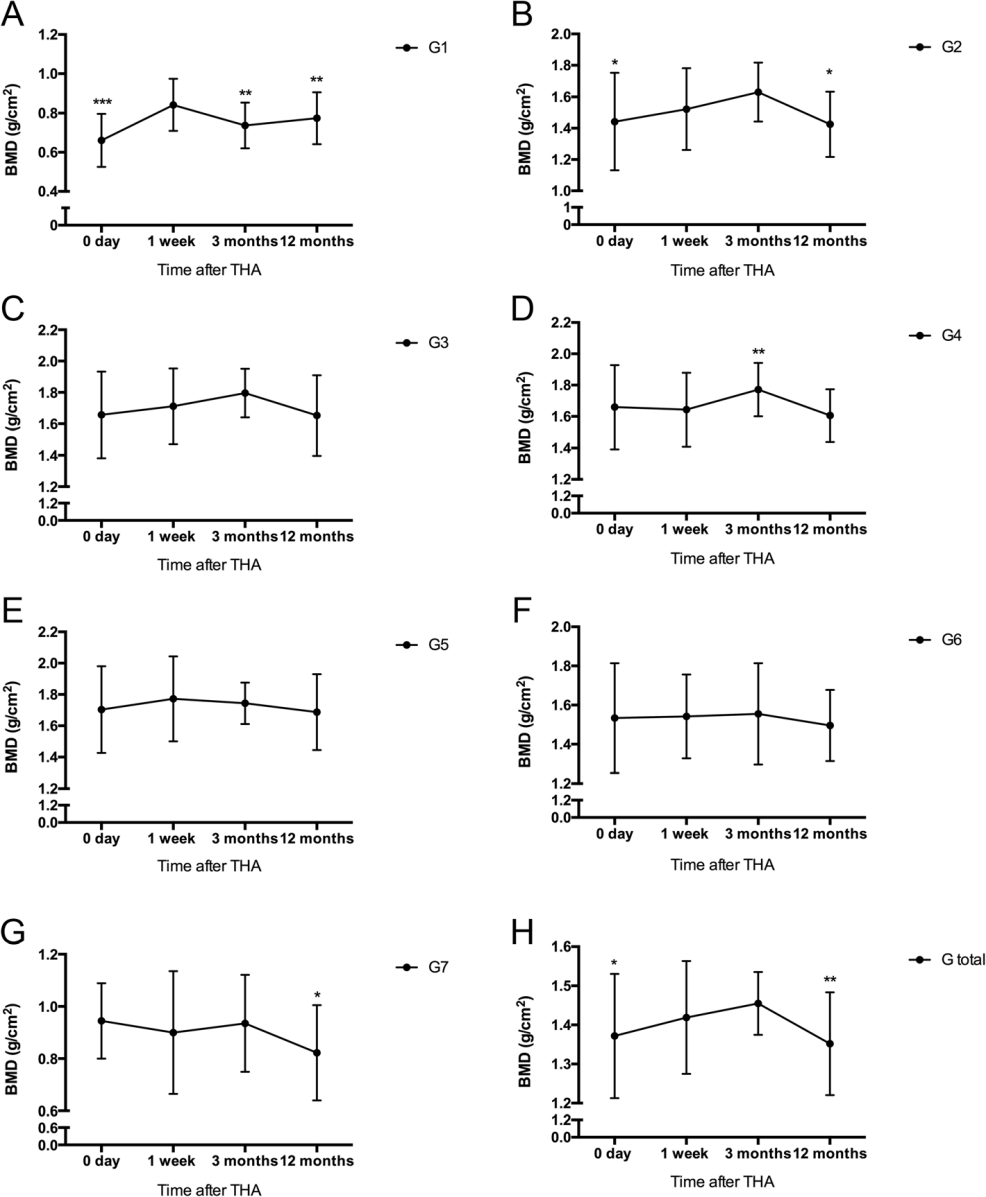
Comparison of the periprosthetic BMD of 7 Gruen zones and total Gruen zone between the different time points during the follow-up period. ^*^ indicated *p* < 0.05, ^**^ indicated *p* < 0.01, and ^***^ indicated *p* < 0.001, when compared with the values measured 1 week after total hip arthroplasty. Comparison of the periprosthetic BMD of Gruen zone 1 to Gruen zone 7 (**a** to **g**) and total Gruen zone (**h**) between the different time points during the follow-up period. * indicated *p*<0.05, ** indicated *p*<0.01, and *** indicated *p*<0.001, when compared with the values measured one week after total hip arthroplasty

In the first postoperative week, we found the periprosthetic BMD data were increased in Gruen zone 1 (+ 27.3%; *p* = 0.000; Fig. 2a), Gruen zone 2 (+ 5.5%; *p* = 0.034; Fig. 2b), and total Gruen zone (+ 3.5%; *p* = 0.021; Fig. 2h) compared with the preoperative data. In the first postoperative year, we found the periprosthetic BMD data in total Gruen zone and all the 7 Gruen zones were decreased compared with the data measured 1 week after surgery. We found significant differences with regard to the data of Gruen zone 1 (− 8.0%; *p* = 0.003; Fig. 2a), Gruen zone 2 (− 6.3%, *p* = 0.033, Fig. 2b), Gruen zone 7 (− 8.6%; *p* = 0.015; Fig. 2g), and total Gruen zone (− 4.7%; *p* = 0.007; Fig. 2h). No significant change was found in Gruen zone 3, 4, 5, and 6 during the 1st postoperative year (Fig. 2c–f).

**Fig. 3:**
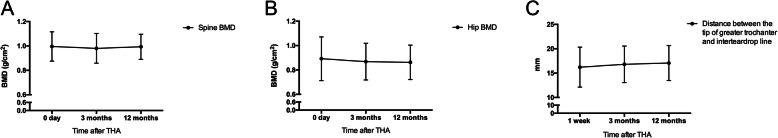
Comparison of the spine (**a**) and hip BMD (**b**), and inferior displacement of the femoral stem (**c**) between the different time points during the follow-up period

**Fig. 4:**
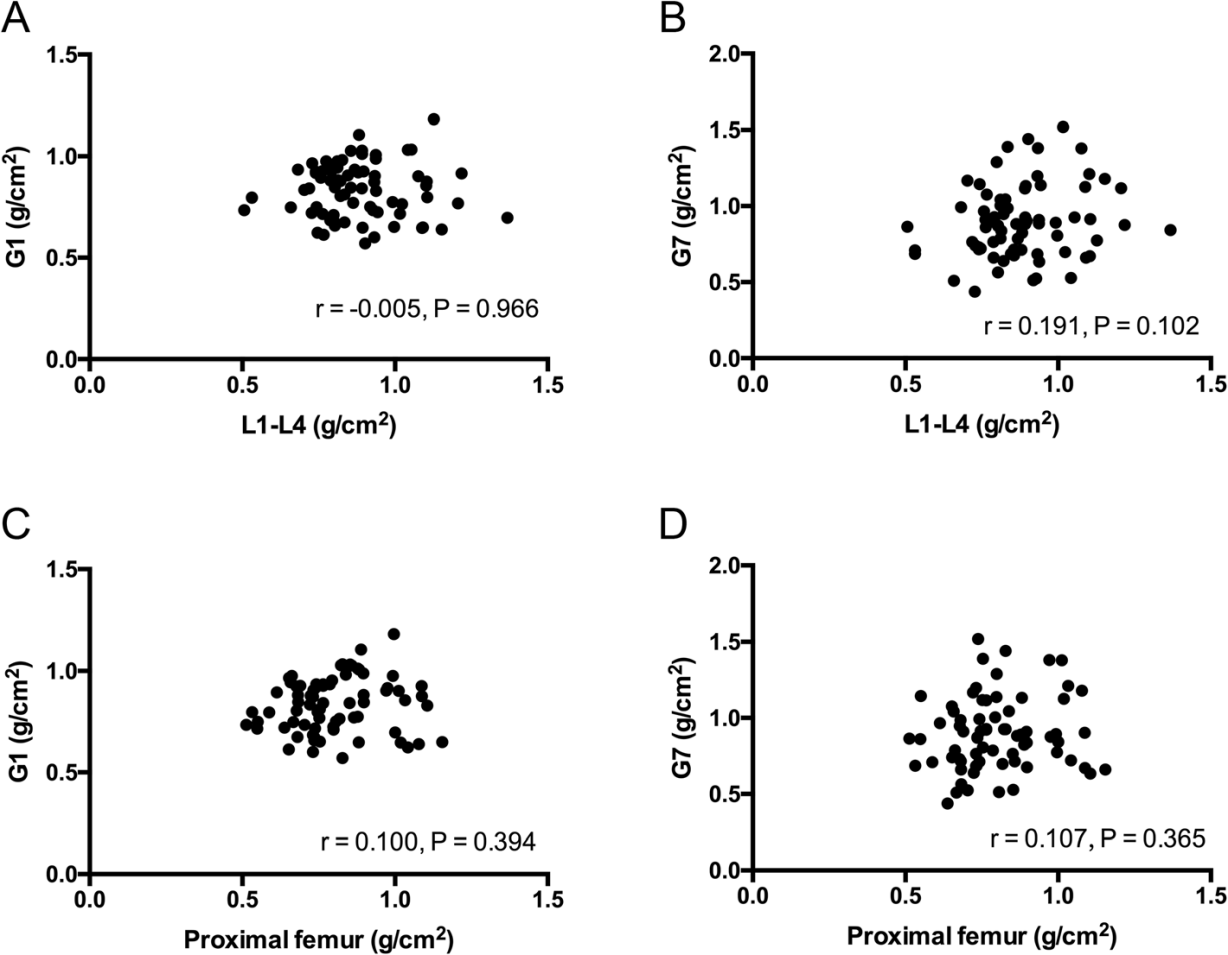
The correlation between preoperative BMD of hip/spine and BMD of Gruen zone 1 (**a**, **c**) /7 (**b**, **d**) measured 1 week after surgery were evaluated by calculation of Pearson correlation coefficient

We did not find a significant change in the BMD of the hip or spine during the period of follow-up (Fig. 3a–b). Correlation was measured by Pearson correlation coefficient. There was no statistically significant difference between the preoperative BMD of hip/spine and the BMD of Gruen zone 1 measured 1 week after THA. The correlation coefficients (r) of spine and total hip BMD were − 0.005 and − 0.1, respectively (Fig. 4a, c). Similar results were also found in Gruen zone 7, with correlation coefficients of 0.191 and 0.107 for spine and total hip BMD, respectively (Fig. 4b, d). None of the patients showed radiographic signs of component loosening or periprosthetic osteolysis. Compared with the baseline, there was no significant difference with regard to the inferior displacement of the femoral stem during the follow-up period (Fig. 3c).

### Periprosthetic bone loss

**Fig. 5:**
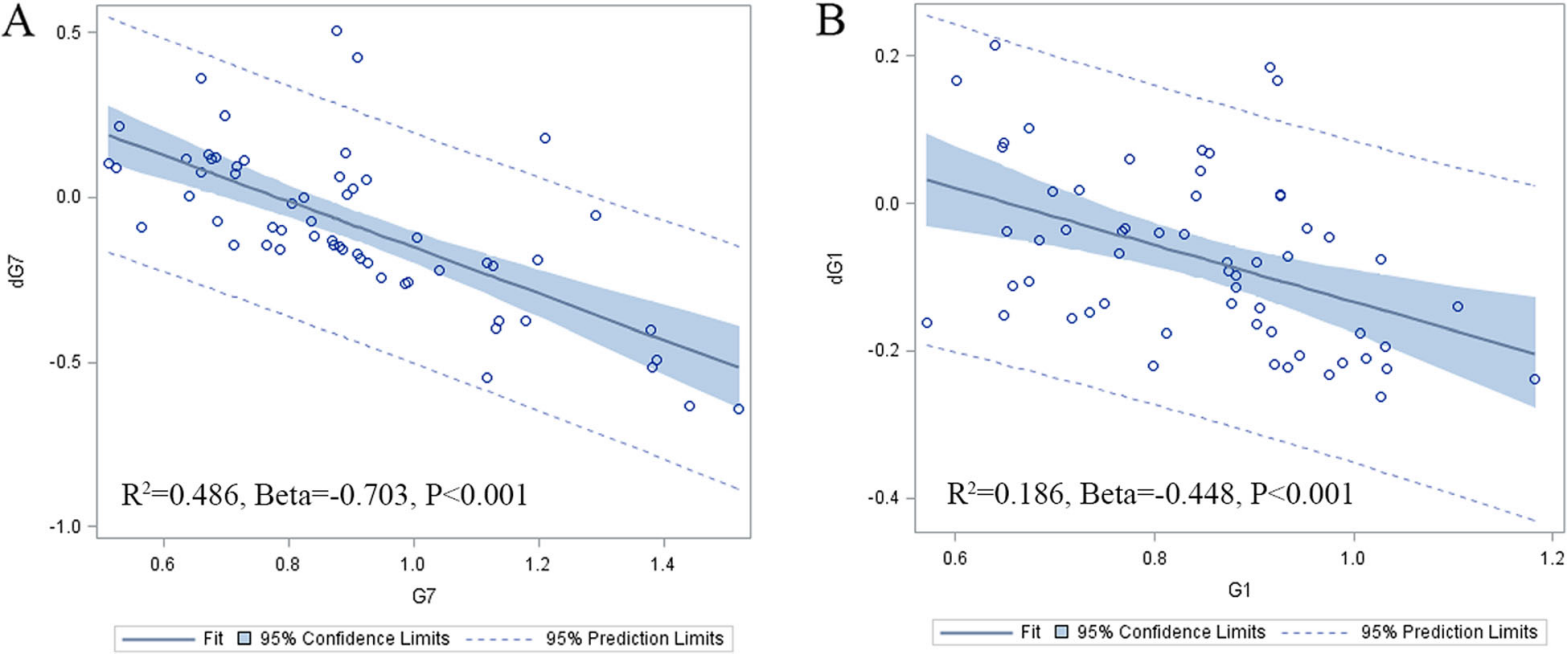
The BMD of Gruen zone 7 (**a**) and 1 (**b**) measured 1 week after surgery were also analyzed for the value as a predictor for BMD changes of Gruen zone 7 and 1 in the first postoperative year by using a multiple linear model with the calculation of the coefficients of determination (R^2^), respectively. Other variables enrolled in the multiple linear analysis included sex, age, BMI, primary diagnosis, and preoperative BMD of hip/spine

Based on the multiple linear regression analysis, we illustrated the BMD of Gruen zone 7 measured 1 week after THA, which was the predictive data of bone loss in Gruen zone 7 for the period of 12 months after THA (Fig. 5a). According to the standardized coefficients, the bone loss in Gruen zone 7 at the end of the follow-up period was negatively affected by BMD of Gruen zone 7 measured 1 week after THA. R^2^ suggested the degree of the aforementioned variation which could explain 48.6% variation of bone loss in Gruen zone 7. Similar results were also found in Gruen zone 1 (Fig. 5b). No significant correlation was found with regard to age and immediate postoperative periprosthetic BMD of Gruen zone 1 (*r* = 0.078; *p* = 0.541) or Gruen zone 7 (*r* = − 0.106; *p* = 0.405). Female patients didn’t show lower immediate postoperative periprosthetic BMD of Gruen zone 1 (0.848 g/cm^2^ vs 0.836 g/cm^2^; *p* = 0.714) and Gruen zone 7 (0.854 g/cm^2^ vs 0.946 g/cm^2^; *p* = 0.115) than male patients.

## Discussion

In the present study, we found that there was significant decrease of periprosthetic BMD in the proximal femur in the first postoperative year. After the first operative year, the BMD of total Gruen zone and the 7 Gruen zones was larger than the least significant change of the hip, spine, and periprosthetic Gruen zones. Messina et al. [14] reported the same biological change. Previous studies showed that the periprosthetic BMD of Gruen zone1 and Gruen zone 7 decreased by 5–10% in the first operative 2 years [11, 15, 16]. Stress-shielding phenomenon was the main determinant of postoperative periprosthetic bone loss after patient’s normal physical activity has recovered [5]. According to the Wolf’s principle, bone mass increases in the areas under loading and decreases in the unloaded regions [17]. In a finite element analysis, Arachchi et al. [18] found the femur was loaded more distally (Gruen zone 3 and Gruen zone 5) with stress bypassing the proximal femur (Gruen zone 1 and Gruen zone 7) after cementless THA. Thus, the decreases of periprosthetic BMD of Gruen zone 1 and Gruen zone 7 were the common findings after cementless THA. In addition to stress-shielding, disuse atrophy, femoral canal preparation, and canal-filling design of the stem also contributed to the short-term postoperative periprosthetic bone loss [19]. Maybe, it is a reasonable explanation for the significant decrease in postoperative BMD of Gruen zone 2 (lateromedial region), as we found in the present study.

We also found the periprosthetic BMD of Gruen zone 1, Gruen zone 2, and total Gruen zone was significantly increased 1 week after THA, compared with the preoperative BMD. Hall et al. [20] and Kröger et al. [21] also found the periprosthetic BMD of 7 Gruen zones was apparently increased immediately after cementless THA. Although the origin of this phenomenon was not entirely clear, the changes are mostly likely the result of compression of cancellous bone when preparing the femoral canal and implanting the femoral prosthesis with the press-fit technique [21]. In consideration of the bias caused by the surgery and implant itself, we suggest that periprosthetic BMD measured 1 week after THA is suitable to serve as the baseline of the postoperative periprosthetic BMD changes.

As we mentioned before, systematic status of bone mass has an influence on the periprosthetic bone loss after THA. Several previous studies proposed that the preoperative BMD of the hip, spine, and radius is the independent factors in predicting the postoperative bone loss [10, 11, 15, 22]. Patients with normal bone showed less bone loss than osteopenic patients in Gruen zone 1 [15]. Lower preoperative systemic BMD (hip and spine) was associated with higher bone loss in Gruen zone 7 [11]. However, the changes of BMD of hip/spine explain why there is only 15% of variation of bone loss in Gruen zone 7 [11]. The values of BMD of spine and contralateral hip are limited in evaluating the bone mass status of periprosthetic regions in perioperative period. The UK National Osteoporosis Guideline Group didn’t recommend the spine as a suitable site for diagnosis of osteoporosis in older people, because a higher prevalence of degenerative changes often leads to an overestimation of BMD of the L1-L4 region [23]. Disuse-induced atrophy also leads to lower BMD of the proximal hip [20]. Damborg et al. [24] reported the preoperative BMD of the affected side was 4.3 to 20% lower than that of the contralateral side. In the present study, we did not find a significant difference between the preoperative BMD of hip/spine and the BMD of Gruen zone 1 measured immediately after THA. Similar results were also observed in Gruen zone 7. Taken together, we suggest that the predictive values of preoperative BMD of hip and spine on periprosthetic bone loss after THA may be limited, because they may not ideally reflect the bone mass status of the periprosthetic regions.

In contrast to our original hypothesis, BMD of Gruen zone 1 and 7 measured 1 week after cementless THA showed a negative correlation with the bone loss in Gruen zone 1 and 7 according to the multiple linear regression analysis. To the best of our knowledge, the relationship between the immediate postoperative periprosthetic BMD and long-term periprosthetic BMD has not yet been investigated before. The underlying mechanism of the former-mentioned phenomenon was still unclear. As we mentioned before, the increase in periprosthetic BMD of the proximal femur measured 1 week after cementless THA is mostly likely the result of the compression of cancellous bone, because the inherent structure of the trabecular bone is destroyed and displaced during the preparation of the femoral canal. The trabecular bone becomes granular shaped and is located mostly in the interface between the implant and host bone after implantation of the femoral prosthesis. We propose that this situation is quite similar to the autogenous cancellous bone grafting. The previous studies have found that there is marked bone resorption after autogenous bone grafting, resulting in a delayed union and nonunion [25, 26]. In the early phase of autogenous cancellous bone transplantation, the necrotic bone is slowly eliminated by macrophages and becomes revascularized, which is followed by formation of new bone by accumulated hematopoietic cells within the transplanted bone [27, 28]. This process leads to complete resorption and replacement of the bone grafts before the bone remodeling was finished [29]. We suppose that it is a reasonable explanation that patients who have high periprosthetic BMD measured in immediate postoperative period are accompanied with advanced periprosthetic bone loss in the early stage of periprosthetic bone remodeling. We also wonder whether age and gender contribute to this phenomenon, because male and younger patients have more postoperative daily living activities that accelerate periprosthetic bone remolding [30]. However, we failed to test this hypothesis, because male and younger patients didn’t show higher immediate postoperative periprosthetic BMD of Gruen zone 1 and Gruen zone 7. Overall, we admit that the mechanism may be more complicated. Other factors, such as stress shielding and aseptic inflammation, may also be involved. Future studies are needed for exploration of this issue. Taken together, we suggested that higher periprosthetic BMD measured in immediate postoperative period may not guarantee a less periprosthetic bone loss in the proximal femur after cementless THA.

The present study was subjected to some limitations. First, it is a single-center, retrospective study with relatively small sample size. The potential influences of bias caused by patient enrollment, data collection, and analysis are inevitable. Some data (i.e., patient weight and height) are missing due to the retrospective design. Nonetheless, the findings are compelling and consistent with the previous reports. Second, the follow-up period is relatively short. However, the changes in the first operative year are clinically relevant, because the initial periprosthetic bone remodeling process is mainly completed in the first 12 to 24 postoperative months [31, 32]. Thus, a 1- to 2-year follow-up is generally adequate for the evaluation of the early-stage periprosthetic bone remodeling [33]. Further studies with a prolonged follow-up period are necessary. Lastly, the results of the present study did not permit us to confirm the threshold values of BMD of Gruen 1 and 7 measured immediately after THA, which indicates a higher risk of periprosthetic bone loss than that of another population. Future studies with larger samples and prospective design are needed for investigation of this issue.

## Conclusion

In summary, the results of the present study indicated that there were marked decreases in the periprosthetic BMD of the proximal femur at the first postoperative year. The predictive values of preoperative BMD of hip and spine on periprosthetic bone loss after THA are limited. Higher periprosthetic BMD measured in immediate postoperative period may not guarantee less periprosthetic bone loss in the proximal femur after cementless THA.

## Data Availability

The datasets used and/or analyzed during the current study are available from the corresponding author on reasonable request.

## Abbreviations

BMD: Bone mineral density
DEXA: Dual-energy x-ray absorptiometry
LSC: Least significant change
THA: Total hip arthroplasty
